# Mindfulness-based intervention improves residual negative symptoms and cognitive impairment in schizophrenia: a randomized controlled follow-up study

**DOI:** 10.1017/S0033291721002944

**Published:** 2023-03

**Authors:** Hui Shen, Li Zhang, Yuhuan Li, Denise Zheng, Lizhao Du, Feikang Xu, Chuchen Xu, Yan Liu, Jie Shen, Zezhi Li, Donghong Cui

**Affiliations:** 1 Shanghai Mental Health Center, Shanghai Jiao Tong University School of Medicine, Shanghai, China; 2 Qingdao Mental Health Center, Qingdao University, Qingdao, China; 3 McGovern Medical School, The University of Texas Health Science Center at Houston, Houston, Texas, USA; 4 Shanghai Med-X Engineering Research Center, School of Biomedical Engineering, Shanghai Jiao Tong University, Shanghai, China; 5 Department of Psychiatry, The Affiliated Brain Hospital of Guangzhou Medical University, Guangzhou, China; 6 Department of Neurology, Ren Ji Hospital, Shanghai Jiao Tong University School of Medicine, Shanghai, China; 7 Shanghai Key Laboratory of Psychotic Disorders, Shanghai, China; 8 Brain Science and Technology Research Center, Shanghai Jiao Tong University, Shanghai, China

**Keywords:** Cognitive impairment, mindfulness, negative symptoms, schizophrenia

## Abstract

**Background:**

Residual negative symptoms and cognitive impairment are common for chronic schizophrenia patients. The aim of this study was to investigate the efficacy of a mindfulness-based intervention (MBI) on negative and cognitive symptoms of schizophrenia patients with residual negative symptoms.

**Methods:**

In this 6-week, randomized, single-blind, controlled study, a total of 100 schizophrenia patients with residual negative symptoms were randomly assigned to the MBI or control group. The 6-week MBI group and the control group with general rehabilitation programs maintained their original antipsychotic treatments. The scores for the Positive and Negative Syndrome Scale (PANSS), the Repeatable Battery for the Assessment of Neuropsychological Status (RBANS), and the Symptom Checklist 90 (SCL-90) were recorded at baseline and week 6 to assess psychotic symptoms, cognitive performance, and emotional state, respectively.

**Results:**

Compared with general rehabilitation programs, MBI alleviated the PANSS-negative subscore, general psychopathology subscore, and PANSS total score in schizophrenia patients with residual negative symptoms (*F* = 33.77, *p*_Bonferroni_ < 0.001; *F* = 42.01, *p*_Bonferroni_ < 0.001; *F* = 52.41, *p*_Bonferroni_ < 0.001, respectively). Furthermore, MBI improved RBANS total score and immediate memory subscore (*F* = 8.80, *p*_Bonferroni_ = 0.024; *F* = 11.37, *p*_Bonferroni_ = 0.006), as well as SCL-90 total score in schizophrenia patients with residual negative symptoms (*F* = 18.39, *p*_Bonferroni_ < 0.001).

**Conclusions:**

Our results demonstrate that MBI helps schizophrenia patients with residual negative symptoms improve clinical symptoms including negative symptom, general psychopathology symptom, and cognitive impairment.

**Trial registration:**

ChiCTR2100043803.

## Introduction

Schizophrenia is a severe, chronic psychiatric disorder that affects 1% of the population and is characterized by psychiatric symptoms and cognitive impairment (Su et al., 2021; Zhu et al., 2020). Although antipsychotic agents are available for treatment, a substantial number of patients with schizophrenia fail to respond to antipsychotics or still have residual symptoms throughout their course of illness, which leads to worse prognoses (Bobes, Arango, Garcia-Garcia, & Rejas, 2010; Kahn et al., 2015; Millan, Fone, Steckler, & Horan, 2014). For example, Schennach et al. reported that 94% of the patients in remission at discharge had at least one residual symptom, 86% had two, 77% had three, and 69% of the patients suffered from at least four residual symptoms. Additionally, the residual symptoms increased the risk of disease recurrence in the following year (Schennach et al., 2015). The most common residual symptoms were blunted affect (49%), conceptual disorganization (42%), and social withdrawal (40%) (Schennach et al., 2015). Even 50% of first-episode schizophrenia patients had residual negative symptoms after remission of 5 years (An der Heiden, Leber, & Häfner, 2016). Another 15-year, prospective, follow-up study revealed that cognitive impairment in first-episode schizophrenia patients persisted for 15 years (Albus et al., 2019). Together, these three studies suggest that residual negative symptoms and cognitive impairment exist throughout almost the entire course of schizophrenia, which consequently makes it difficult for patients to use complex cognitive strategies to deal with problems, thus increases the burden on individuals, families, and society (Charlson et al., 2018). Therefore, comprehensive treatment strategies should be emphasized in patients with schizophrenia, especially in chronic patients who have residual negative symptoms and cognitive impairment.

Current National Institute for Health and Care Excellence (NICE) guidelines from the UK recommend treating schizophrenia by combining antipsychotics with psychological-related intervention. Psychosocial intervention has gradually become a critical adjuvant therapy for antipsychotic agents including cognitive-behavioral therapy (CBT), family interventions, and supported employment programs (Norman, Lecomte, Addington, & Anderson, 2017). These interventions attenuate psychiatric symptoms, help patients acquire social skills, improve cognitive function, and reduce the rate of recurrence and hospitalizations (Norman et al., 2017; Pfammatter, Junghan, & Brenner, 2006). In recent years, increasing attention has been given to meditation, one of the most promising psychosocial-related interventions that has developed into a kind of psychotherapy technology (Shen, Chen, & Cui, 2020; Simkin & Black, 2014). Mindfulness-based intervention (MBI) is the most widely used meditation technique and includes mindfulness-based stress reduction (MBSR), mindfulness-based cognitive therapy (MBCT), dialectical behavior therapy (DBT), acceptance and commitment therapy (ACT), and mindfulness-based relapse prevention (Shen et al., 2020; Simkin & Black, 2014). Preliminary research has shown that MBI helped alleviate anxiety, sleep, and boredom in hospitalized patients with acute psychiatric disorders (Mistler, Ben-Zeev, Carpenter-Song, Brunette, & Friedman, 2017), as well as helped regulate emotions in outpatients with schizophrenia (Tabak, Horan, & Green, 2015). MBI can also improve a variety of clinical presentations of schizophrenia patients (Hodann-Caudevilla, Díaz-Silveira, Burgos-Julián, & Santed, 2020), including positive and negative symptoms (Chien, Bressington, Yip, & Karatzias, 2017; Chien, Cheng, McMaster, Yip, & Wong, 2019; Lee, 2019; Wang, Chien, Yip, & Karatzias, 2016), insight into illness/treatment, and duration of hospitalization (Chien & Lee, 2013; Chien et al., 2017, 2019; Wang et al., 2016).

The effect of MBI on cognitive impairment in patients with schizophrenia, however, has not been investigated. It is well established that cognitive impairment is a core symptom of schizophrenia in patients with acute stage or stable chronic schizophrenia (Huang et al., 2020; Kahn & Keefe, 2013; Li et al., 2020; Zhou et al., 2020) and is also the main goal of treatment in clinical practice (Harvey, 2009). Several studies have found that MBI can improve the cognitive function of healthy adults, such as improving cognitive flexibility and fluency, increasing creativity (Henriksen, Richardson, & Shack, 2020) and attention (Basso, McHale, Ende, Oberlin, & Suzuki, 2019; Ziegler et al., 2019), and improving visual-spatial processing, working memory, and executive function (Fabio & Towey, 2018; Zeidan, Johnson, Diamond, David, & Goolkasian, 2010). In addition, recent system reviews suggested that MBI can improve cognitive function, including attention, executive function, and working memory. The integration of meditation practice into a broader cognitive remedial and therapeutic education program may help to improve the cognitive function of patients with cognitive impairment, especially in the field of psychiatry (Bulzacka, Lavault, Pelissolo, & Bagnis Isnard, 2018). This provides a new direction for the treatment of cognitive impairment in patients with mental disorders. The latest research has found that a comprehensive psychological method including mindfulness training, psychological education, and functional repair reduced difficulties in the cognitive domain of the Functioning Assessment Short Test (FAST) in patients with bipolar disorder (Valls et al., 2021). However, it remains unclear whether MBI can improve the residual symptom of cognitive impairment in patients with chronic schizophrenia.

Therefore, to the best of our knowledge, this 6-week, randomized controlled trial (RCT) was the first to investigate the effect of MBI on the improvement of residual symptoms, such as negative symptoms and cognitive impairment, in remitted, chronic, schizophrenia patients. We hypothesized that MBI could improve residual symptoms of negative symptoms and cognitive impairment compared to traditional rehabilitation programs in remitted, chronic, schizophrenia patients.

## Methods

### Participants

This study was approved by the Institutional Ethics Committee of Shanghai Mental Health Center. All participants signed written informed consents. Patients with schizophrenia were recruited from the rehabilitation ward of Shanghai Mental Health Center from 1 October 2020 to 30 November 2020. The clinical trial registration number is ChiCTR2100043803.

The inclusion criteria were as follows: (1) Han Chinese population; (2) age ⩾18 years, (3) education level ⩾6 years; (4) satisfied with the diagnostic criteria for schizophrenia based on the Diagnostic and Statistical Manual of Mental Disorders; (5) patients with chronic schizophrenia residing in the rehabilitation ward, without relapse in the past 6 months; (6) residual negative symptoms, with at least one item ⩾2 on the negative subscale of PANSS (N1–N7) (Schennach et al., 2015); (7) taking second generation antipsychotics.

The exclusion criteria were as follows: (1) having severe physical diseases such as cardiovascular, lung, liver, kidney, and hematopoietic diseases; (2) comorbid other Axis I diagnosis; (3) having alcohol or substance abuse/dependence; (4) having mental retardation and/or severe cognitive impairment; (5) electroconvulsive or rTMS therapy in past 3 months. Alcohol or drug abuse/dependence patients are determined through medical records and self-reported alcohol/drug use (Lv et al., 2020; Zhou et al., 2021). Patients with mental retardation were interviewed and excluded by clinicians according to their medical history and IQ score (WAIS < 70). The Mini mental state examination (MMSE) assessment was used, and patients whose MMSE < 24 were identified as severe cognitive impairment (Albanna et al., 2017; Lopez, Charter, Mostafavi, Nibut, & Smith, 2005).

**Fig. 1.**
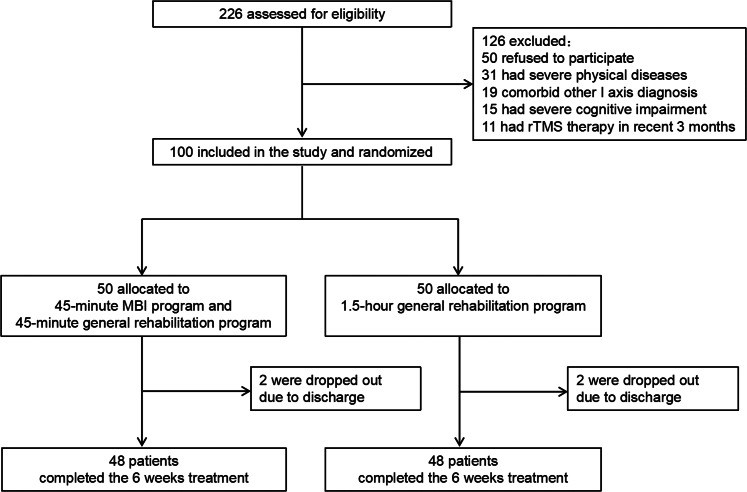
Study flowchart.

A total of 226 inpatients with schizophrenia were screened in this clinical trial. Among them, 50 patients did not sign informed consent forms, 31 patients had severe physical diseases, 19 patients had other comorbid Axis 1 diagnoses, 15 patients had severe cognitive impairment, and 11 patients had rTMS therapy in the past 3 months; thus, they were all excluded. A final total of 100 patients were included in this study. There were 68 males and 32 females. The average age was 59.50 ± 8.11 years old. The CONSORT diagram of the recruitment process in this study is shown in Fig. 1.

### Clinical interview and assessments

Patients were interviewed by psychiatrists using the Structured Clinical Interview for DSM-IV (SCID-I/P). In this study, demographic and clinical data were collected such as age, sex, education, family history, age of onset, illness duration, and medicine use. MMSE was used to screen severe cognitive impairment. The psychiatric symptoms were evaluated using the Positive and Negative Syndrome Scale (PANSS) (Kay, Fiszbein, & Opler, 1987). Cognitive performance of patients with schizophrenia was evaluated using the Repeatable Battery for the Assessment of Neuropsychological Status (RBANS) (Randolph, Tierney, Mohr, & Chase, 1998). Emotional state was evaluated using the anxiety and depression subscales from the Symptom Checklist-90 (SCL-90) (Hunter et al., 2005). All psychiatrists were trained in the assessments of PANSS, RBANS, and SCL-90 before the start of the study. The inter-rater correlation coefficients of PANSS and RBANS were more than 0.8.

### Randomization, masking, and procedures

Once the recruitment process was completed, all eligible patients were randomized 1:1 for either the intervention or the control group. Randomization was conducted via a computer-generated, random identification number.

Patients in the control group received general rehabilitation. This program happened once a day for 1.5 h from Monday to Friday and lasted for 6 weeks, for a total of 30 workshops. The general rehabilitation program included general health education, reading, painting, manual work, gardening, and daily life skills training.

Patients in the intervention group received 45 min of MBI group programming and 45 min of general rehabilitation programming. The MBI programming happened once a day for 45 min from Monday to Friday and lasted for 6 weeks, for a total of 30 workshops. The 10 MBI topics covered were as follows, with each topic focused on for 3 days: Topic 1: orientation to mindfulness (team building, introduction to mindfulness, posture for mindfulness practice, focus on breathing and staying present); Topic 2: mindfulness and rumination (identify rumination, focus on breathing and staying present, focused awareness of thoughts); Topic 3: cognitive patterns (identify automatic thinking patterns, focused awareness of bodily sensations and thoughts); Topic 4: understanding schizophrenia (introduction to psychiatric symptoms, awareness of breath and sounds, awareness of breath and thoughts); Topic 5: sharing about disease course (peer sharing, encourage mindfulness in life, mindful eating, mindful stretching); Topic 6: identifying emotions during illness process (thoughts, emotions, physical feelings, focusing on both pleasant and annoying events, 3 min breathing session); Topic 7: self-acceptance (acceptance of disease, acceptance of residual symptoms, practice of self-care, practice loving yourself); Topic 8: acceptance of medication (identify adverse drug reactions, body scan, mindful walking); Topic 9: relapse prevention (awareness of symptoms, identify signs of relapse and associated factors, learning effective coping and problem-solving skills); Topic 10: summary and discussion (sharing feelings, reviewing mindfulness practice, group discussion about future problems, encouraging integration of mindfulness into daily life).

All patients maintained their original antipsychotic treatments. Patients who suffered from insomnia or anxiety were treated with benzodiazepines for a limited period of time. Patients who are suffering from extrapyramidal symptoms were treated with trihexyphenidyl over a short period of time. All patients were told to attend a rehabilitation program but did not receive suggestions on the differences and efficacy between the rehabilitation programs and MBI.

All raters were blinded to the patient's intervention group status throughout the study. The assistant who managed the research evaluation schedule for each participant was also blinded to the adjunctive intervention groups. The therapists in charge of the intervention treatment were not blinded. Due to the nature of the intervention, participants could not be blinded to their intervention group assignment, but they were asked not to reveal their assignment to the raters.

Assessments (PANSS, RBANS, and SCL-90) were conducted at baseline and week 6. The primary outcome was the PANSS score at week 6. The secondary outcome was RBANS and SCL-90 score at week 6.

### Statistical analysis

Kolmogorov–Smirnov one-sample test was applied to assess the distribution of the data. The χ^2^test was applied for categorical variables. The analysis of variance (ANOVA) or nonparametric test was applied for continuous variables. To analyze sensitivity, an intent-to-treat analysis was applied, and the principle of last-observation-carrying-forward was conducted to address the missing data.

Repeated-measures analysis of variance (RM ANOVA) was applied to examine each PANSS score, RBANS score, and their respective subscale score, including a between-group factor (intervention and control), within-group factor (baseline and week 6), and group × time interaction. RM ANOVA was also applied to examine the SCL-90 total subscale score and its depression and anxiety subscale score. After performing RM ANOVA, multivariate omnibus test was performed as follow-up, and the individual univariate effects were examined by ANCOVA. If there was no significant effect of group × time interaction, no further statistical test was needed. If the group × time interaction had a significant effect, the group difference was analyzed by ANCOVA at the 6th week, with baseline score, age, education, illness duration, and drug dose of equivalent olanzapine as covariates. Bonferroni correction was conducted for multiple tests. The patients’ dose of antipsychotics was converted into olanzapine equivalents (Leucht, Samara, Heres, & Davis, 2016). All statistical analyses were conducted in PASW Statistics, Version 23.0 (SPSS Inc., Chicago, IL, USA).

## Results

### Demographics and baseline data

As shown in Fig. 1, a total of 226 participants were assessed for eligibility. A final total of 100 patients were included and randomly assigned to either the intervention group or the control group. During the study, two patients in each group dropped out after discharge (4%). Ninety-six patients completed the 6-week trial (48 patients in the intervention group and 48 patients in the control group). All patients continued to receive their previous antipsychotic regimen (unchanged type and dose) during the course of the study. Patients received the antipsychotic agents including clozapine, olanzapine, quetiapine, aripiprazole, ziprasidone, risperidone, amisulpride and paliperidone. Among them, 61 patients were treated with single antipsychotic drug, 39 were treated with two antipsychotics. In addition, 19 patients received benzodiazepines and 18 patients received trihexyphenidyl.

As shown in Table 1, there was no significant difference in demographic data and clinical data (age, sex, marital status, education, family history, frequency of hospitalization, and illness duration) or clinical evaluation (score of PANSS, RBANS, SCL-90) between the two groups at baseline (all *p* > 0.05). The ages range from 35 to 74 years old (35–44 years old: 4 cases; 45–54 years old: 18 cases; 55–64 years old: 50 cases; 65–74: years old: 28 cases), and there was no difference in frequency between two groups. Furthermore, the dose of antipsychotics taken by patients was converted into olanzapine equivalents (Leucht et al., 2016), and there was no significant difference in drug dose of equivalent olanzapine (*p* > 0.05). There was no significant difference in the use of benzodiazepines and trihexyphenidyl between the two groups (all *p* > 0.05).

**Table 1.** Demographic and clinical data of MBI and control groups at baseline

	MBI (*n* = 50)	Control (*n* = 50)	χ^2^, *Z*,or *F* (p)
Age (years)	59.24 ± 9.51	59.76 ± 6.51	0.10 (0.75)
Sex (male/female)	33/17	35/15	0.18 (0.67)
Marital status (married/unmarried/divorce or widowhood)	16/25/9	10/23/17	3.93 (0.14)
Education (years)	9.92 ± 2.24	9.84 ± 2.13	0.03 (0.86)
Family history (no/yes)	38/12	37/13	0.05 (0.82)
Frequency of hospitalization (frequency)	3.64 ± 2.79	3.76 ± 3.00	−0.26 (0.80)
Illness duration (years)	33.04 ± 10.85	33.84 ± 8.24	0.17 (0.68)
Drug dose of equivalent OLZ (mg/d)	13.10 ± 6.43	13.48 ± 5.80	−0.52 (0.60)
Benzodiazepines (no/yes)	41/9	40/10	0.07 (0.80)
Trihexyphenidyl (no/yes)	40/10	42/8	0.27 (0.60)
PANSS total score	56.68 ± 10.38	57.16 ± 9.81	0.06 (0.81)
P subscore	10.34 ± 4.08	10.44 ± 4.04	0.02 (0.90)
N subscore	16.50 ± 3.23	16.70 ± 3.81	0.08 (0.78)
G subscore	29.84 ± 5.29	30.02 ± 4.53	0.03 (0.86)
RBANS total score	80.38 ± 12.17	80.22 ± 10.56	0.01 (0.94)
IM	71.64 ± 16.07	71.96 ± 12.77	0.01 (0.91)
VC	87.10 ± 14.53	86.40 ± 12.09	0.07 (0.79)
Language	88.94 ± 7.40	88.74 ± 7.79	0.02 (0.90)
Attention	94.66 ± 14.29	94.38 ± 12.70	0.01 (0.92)
DM	82.74 ± 15.26	81.80 ± 16.22	0.09 (0.77)
SCL-90 total score	1.60 ± 0.26	1.59 ± 0.23	0.05 (0.83)
Depression subscale	1.67 ± 0.34	1.62 ± 0.30	0.50 (0.48)
Anxiety subscale	1.54 ± 0.27	1.55 ± 0.23	0.06 (0.81)

MBI, mindfulness-based intervention; OLZ, olanzapine; PANSS, Positive and Negative Syndrome Scale; P, positive symptom; N, negative symptom; G, general psychopathology symptom; IM, immediate memory score; VC, visuospatial/construction score; DM, delayed memory score.

### Effect of a mindfulness-based intervention on PANSS score

As shown in Table 2, RM ANOVA demonstrated significant time effect and group × time effect on PANSS total score (Wilks’ *λ F* = 31.45, *p* < 0.001; Wilks’ *λ F* = 49.02, *p* < 0.001), negative symptom subscore (Wilks’ *λ F* = 10.58, *p* = 0.002; Wilks’ *λ F* = 34.29, *p* < 0.001), and general psychopathology subscore (Wilks’ *λ F* = 32.52, *p* < 0.001; Wilks’ *λ F* = 33.64, *p* < 0.001).

**Table 2.** The scores of PANSS, RBANS, SCL-90 at baseline and week 6 in MBI and control groups

	Baseline		Week 6		Group	Time	Group × time	ANOVA
	MBI	Control	MBI	Control	*F*(p)	*F*(p)	*F*(p)	*F*(p)
PANSS total score	56.68 ± 10.38	57.16 ± 9.81	53.06 ± 9.35	57.56 ± 10.07	1.61 (0.207)	31.45 (<0.001)	49.02 (<0.001)	52.41 (<0.001)
P subscore	10.34 ± 4.08	10.44 ± 4.04	10.20 ± 3.86	10.50 ± 4.18	0.06 (0.804)	0.25 (0.619)	1.56 (0.215)	1.51 (0.223)
N subscore	16.50 ± 3.23	16.70 ± 3.81	15.38 ± 3.12	17.02 ± 3.97	1.73 (0.192)	10.58 (0.002)	34.29 (<0.001)	33.77 (<0.001)
G subscore	29.84 ± 5.29	30.02 ± 4.53	27.48 ± 4.49	30.04 ± 4.32	2.26 (0.136)	32.52 (<0.001)	33.64 (<0.001)	42.01 (<0.001)
RBANS total score	80.38 ± 12.17	80.22 ± 10.56	84.26 ± 13.42	80.96 ± 12.36	0.53 (0.469)	20.53 (<0.001)	9.48 (0.003)	8.80 (0.004)
IM	71.64 ± 16.07	71.96 ± 12.77	78.44 ± 17.49	73.40 ± 15.33	0.62 (0.434)	28.84 (<0.001)	12.20 (0.001)	11.37 (0.001)
VC	87.10 ± 14.53	86.40 ± 12.09	89.70 ± 13.84	86.92 ± 12.53	0.47 (0.496)	4.13 (0.045)	1.84 (0.179)	2.03 (0.158)
Language	88.94 ± 7.40	88.74 ± 7.79	90.60 ± 6.87	89.70 ± 6.78	0.17 (0.686)	6.78 (0.011)	0.48 (0.488)	0.66 (0.420)
Attention	94.66 ± 14.29	94.38 ± 12.70	95.10 ± 15.31	94.78 ± 11.80	0.01 (0.910)	0.53 (0.470)	0.001 (0.972)	0.03 (0.868)
DM	82.74 ± 15.26	81.80 ± 16.22	87.20 ± 16.67	82.24 ± 17.06	0.87 (0.353)	9.15 (0.003)	6.16 (0.015)	5.98 (0.016)
SCL-90 total score	1.60 ± 0.26	1.59 ± 0.23	1.44 ± 0.23	1.55 ± 0.20	1.43 (0.234)	38.88 (<0.001)	15.24 (<0.001)	18.39 (<0.001)
Depression subscale	1.67 ± 0.34	1.62 ± 0.30	1.47 ± 0.29	1.59 ± 0.29	0.52 (0.471)	14.84 (<0.001)	7.29 (0.008)	7.65 (0.007)
Anxiety subscale	1.54 ± 0.27	1.55 ± 0.23	1.41 ± 0.27	1.52 ± 0.26	1.63 (0.205)	10.44 (0.002)	3.45 (0.066)	4.71 (0.033)

MBI, mindfulness-based intervention; PANSS, Positive and Negative Syndrome Scale; P, positive symptom; N, negative symptom; G, general psychopathology symptom; IM, immediate memory score; VC, visuospatial/construction score; DM, delayed memory score.

**Fig. 2.**
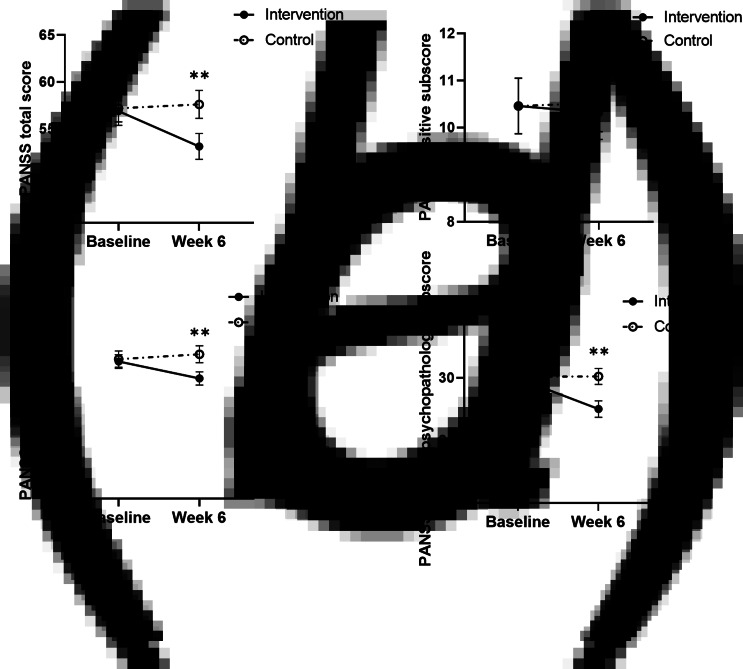
Effect of MBI on PANSS score. (a) ANCOVA showed that PANSS total score was lower in the intervention group than that in control group at week 6 (*F* = 52.41, *p*_Bonferroni_ < 0.001, Cohen's *d* = 0.46). (b) There was no difference in PANSS-positive subscore between two groups at week 6 (*p* > 0.05). (c) ANCOVA showed that PANSS-negative symptom subscore was lower in the intervention group than that in control group at week 6 (*F* = 33.77, *p*_Bonferroni_ < 0.001, Cohen's *d* = 0.46). (d) ANCOVA showed that psychopathology subscore was lower in the intervention than that in control group at week 6 (*F* = 42.01, *p*_Bonferroni_ < 0.001, Cohen's *d* = 0.58). *p < 0.05, **p < 0.01.

ANCOVA was then applied to further investigate the group differences in PANSS total score and its subscale score at week 6, after adjusting for age, education, illness duration, drug dose of equivalent olanzapine, and baseline PANSS score as covariates. As shown in Fig. 2, PANSS total score, negative symptom subscore, and general psychopathology subscore were lower in the intervention group than those in the control group at week 6 (all *p*_Bonferroni_ < 0.001).

### Effect of a mindfulness-based intervention on cognitive function

RM ANOVA was conducted on RBANS total score and each subscale score separately. As shown in Table 2, there were significant time effect and group × time effect on RBANS total score (Wilks’ *λ F* = 20.53, *p* < 0.001; Wilks’ *λ F* = 9.48, *p* = 0.003), immediate memory (Wilks’ *λ F* = 28.84, *p* < 0.001; Wilks’ *λ F* = 12.20, *p* = 0.001), and delayed memory (Wilks’ *λ F* = 9.15, *p* = 0.003; Wilks’ *λ F* = 6.16, *p* = 0.015).

**Fig. 3.**
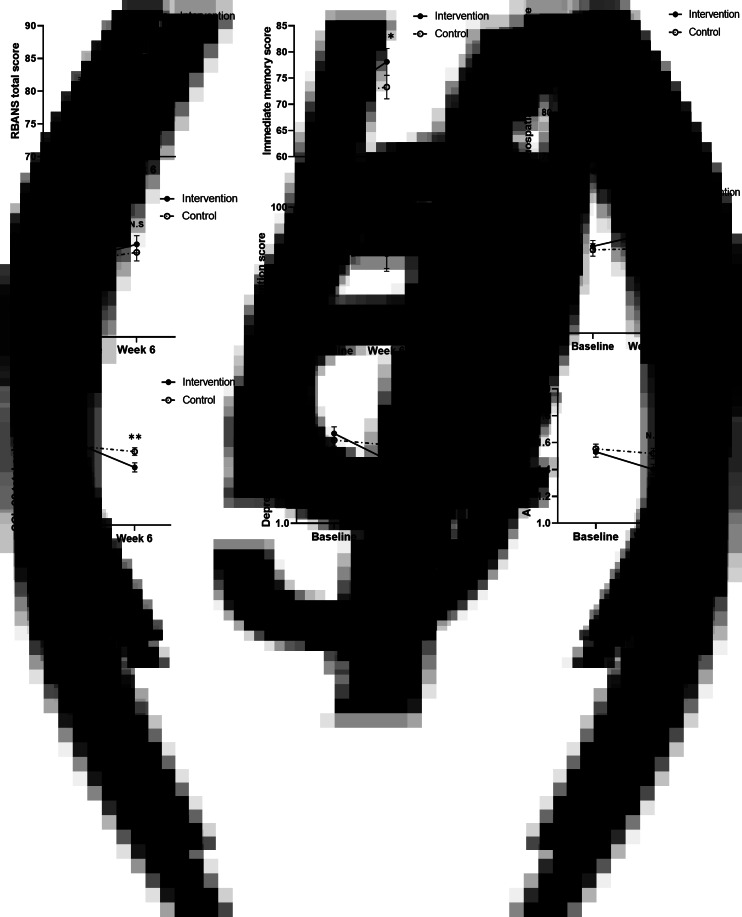
Effect of MBI on RBANS and SCL-90 score. (a–f) ANCOVA showed that RBANS total score and immediate memory score were higher in the intervention group than those in the control group at week 6 (*F* = 8.80, *p*_Bonferroni_ = 0.024, Cohen's *d* = 0.26; *F* = 11.37, *p*_Bonferroni_ = 0.006, Cohen's *d* = 0.31) (a, b), while there was no difference in visuospatial/construction score, language score, attention score, or delayed memory score between two groups at week 6 (*p*_Bonferroni_ > 0.05) (c–f); (g–i) ANCOVA showed that SCL-90 total score was lower in the intervention group than that in the control group at week 6 (*F* = 18.39, *p*_Bonferroni_ < 0.001, Cohen's *d* = 0.51) (g), while there was no difference in SCL-90 depression subscore or anxiety subscore between two groups at week 6 (*p*_Bonferroni_ > 0.05) (h, i). *p < 0.05, **p < 0.01.

ANCOVA was then used to further investigate the group differences in RBANS total score, immediate memory, and delayed memory at week 6, after adjusting covariates. As shown in Figs 3a–f, the RBANS total score, immediate memory, and delayed memory were higher in the intervention group than those in the control group at week 6 (*p* = 0.004, Cohen's *d* = 0.26; *p* = 0.001, Cohen's *d* = 0.31; *p* = 0.016, Cohen's *d* = 0.29, respectively). However, only RBANS total score and immediate memory had a significant difference after Bonferroni correction (*p*_Bonferroni_ = 0.024, *p*_Bonferroni_ = 0.006).

### Effect of a mindfulness-based intervention on SCL-90

As shown in Table 2, RM ANOVA showed significant time effect and group × time effect on SCL-90 total subscale score (Wilks’ *λ F* = 38.88, *p* < 0.001; Wilks’ *λ F* = 15.24, *p* < 0.001) and its depression subscale score (Wilks’ *λ F* = 14.84, *p* < 0.001; Wilks’ *λ F* = 7.29, *p* = 0.008).

ANCOVA was then applied to further investigate the group differences in SCL-90 total subscale score and its depression subscale score at week 6, after adjusting covariates. As shown in Figs 3g–i, the intervention group had a lower SCL-90 total subscale score, depression subscale score, and anxiety subscale score than the control group at week 6 (*p* < 0.001, Cohen's *d* = 0.51; *p* = 0.007, Cohen's *d* = 0.41; *p* = 0.033, Cohen's *d* = 0.42, respectively). However, after Bonferroni correction, SCL-90 total score was still significant (*p*_Bonferroni_ < 0.001).

## Discussion

To the best of our knowledge, this was the first time a study investigated the effects of MBI on negative symptoms and cognitive impairment of schizophrenia patients with residual negative symptoms. The main results of this study were as follows: (1) MBI can improve residual negative symptoms and general psychopathological symptoms in schizophrenia patients; (2) MBI can significantly improve cognitive impairment in schizophrenia patients with residual negative symptoms; (3) MBI can alleviate SCL-90 score in schizophrenia patients with residual negative symptoms.

Previous studies reported that MBI or mindfulness-based psychoeducation showed some efficacy in reducing negative symptoms of schizophrenia. For example, Lee et al. recruited chronic schizophrenia patients to undergo an eight-session (1.5 h each) MBI program guided by therapists once a week. They found that MBI mitigated the severity of negative and general psychopathology symptoms (Lee, 2019). Other studies recruited outpatients with schizophrenia for 24-week, mindfulness-based psychoeducation (2 h program guided by therapists biweekly) and discovered that MBI improved both negative and positive symptoms (Chien et al., 2017, 2019; Wang et al., 2016). The latest systematic review and meta-analysis of acceptance- and mindfulness-based therapies for people with a psychotic or schizophrenia spectrum disorder showed effects on negative symptoms, but no significant effects for positive symptoms (Jansen, Gleeson, Bendall, Rice, & Alvarez-Jimenez, 2020). However, most prior studies had weekly or biweekly MBI sessions guided by therapists, and patients did their homework by themselves the rest of the time. Most schizophrenia patients have negative symptoms, which may lead to a lack of initiative in meditation practice. Therefore, it may be difficult for patients to practice mindfulness on their own accord, and the quality of mindfulness practice cannot be guaranteed.

CBT is recommended as one of the effective treatments for schizophrenia (Norman et al., 2017). Some of the current new forms of CBT include various mindfulness-based interventions (MBIs), including MBCT, MBSR, ACT, and DBT (Feliu-Soler et al., 2018), which have been initially applied in patients with schizophrenia and have obtained positive results (Hodann-Caudevilla et al., 2020; Jansen et al., 2020). Based on the above foundation, this study based on the disease characteristics of patients with chronic schizophrenia, combined with mindfulness and CBT, compiled this MBI course. The course includes mindfulness attention training, mindfulness awareness, description of thoughts, emotional and physical feelings, and cognitive reconstruction to help patients better accept residual symptoms and medications, and encourage patients to self-acceptance and self-compassion.

In the present study, we applied MBI in the treatment of schizophrenia patients with residual negative symptoms. Besides intensive teaching, the course also included intensive practice guided by therapists five times a week, which may better ensure the quality of mindfulness practice. We discovered that MBI can significantly improve residual negative and general psychopathology symptoms in these specific individuals. Although the mechanism of MBI improving negative symptoms of schizophrenia is unknown, there may be psychological and biological explanations. For a psychological explanation, several negative symptoms are associated with metacognition, such as blunted affect, poor rapport, and alogia (Austin et al., 2019). Mindfulness may improve metacognition, increase positive self-empathy, and promote recovery in psychosis (Hochheiser, Lundin, & Lysaker, 2020). Mindfulness also contributes to the increase of self-compassion and regulation of emotion (Jansen et al., 2020; Tabak et al., 2015). Campellone elucidated a new potential mechanism for negative symptoms and poor functional outcome: defeatist performance beliefs. In other words, negative thoughts about one's ability to successfully perform goal-directed behavior can prevent behavior initiation and engagement (Campellone, Sanchez, & Kring, 2016). Thus, mindfulness could help improve negative symptoms because it helps patients learn to observe negative thoughts and feelings in a nonjudgmental way, cultivates an appreciation of natural mentality, and establishes a more positive attitude.

A possible biological explanation for why MBI improved negative symptoms of schizophrenia is as follows. Accumulating evidence has shown that exacerbations of negative symptoms were associated with elevated levels of cytokines, such as interleukin (IL)-6, tumor necrosis factor-*α*, IL-1*β*, interferon-*γ*, IL-12, and transforming growth factor-*β* (Momtazmanesh, Zare-Shahabadi, & Rezaei, 2019). MBI has been shown to modulate the levels of cytokines (Shen et al., 2020). In addition, neuroimaging evidence has shown that decreased activity in the prefrontal cortex was associated with negative symptoms of schizophrenia, which can be improved by enhanced prefrontal cortex activity (Micoulaud Franchi, Quiles, Belzeaux, Adida, & Azorin, 2015). MBI has been shown to activate the prefrontal cortex (Sperduti, Martinelli, & Piolino, 2012). Lastly, decreased activity of the cingulate cortex was closely related to negative symptoms (Bersani et al., 2014). MBI can enhance the activity of the cingulate cortex (Fox et al., 2014). Further studies should be conducted to confirm these possible mechanisms.

It is well-established that cognitive impairment is one of the core symptoms of schizophrenia and that it is closely related to negative symptoms (Lin et al., 2013). Currently, few studies have investigated the effect of mindfulness intervention on cognitive function in patients with schizophrenia. A recent RCT study showed that adjunctive treatment (including psychoeducation, mindfulness training, and functional remediation) represented a promising cost-effective therapy to improve psychosocial functioning and residual depressive symptoms and reduce difficulties in the cognitive domain of FAST in patients suffering from bipolar disorders (Valls et al., 2021). Another qualitative review showed that MBI enhanced positive effect, reduced symptoms of anxiety and depression, and improved memory and executive function of the elderly (Hazlett-Stevens, Singer, & Chong, 2019). Consistently, our study found that MBI can significantly improve cognitive function, especially immediate memory, in schizophrenia patients with residual negative symptoms. There are several explanations that could elucidate the mechanism of how MBI impacts cognitive function. First, mindfulness can help patients learn to observe negative thoughts and feelings in a nunjudged manner, increase self-sympathy, and develop a more positive attitude (Hochheiser et al., 2020). Relevant electrophysiological studies suggested that mindfulness attention training can improve sustained attention and working memory of healthy young people by improving key neural signatures of attentional control (frontal theta inter-trial coherence and parietal P3b latency) (Ziegler et al., 2019). Neuroimaging studies also revealed a decreased anticorrelation between default mode network (DMN) and central executive network, which is associated with cognitive impairment (Bauer et al., 2020; Whitfield-Gabrieli & Ford, 2012). Meditation also modulates brain network integration and increases DMN anticorrelations in schizophrenia patients, which improves cognitive functions such as attention and working memory (Bauer et al., 2020).

The effect of MBI on positive symptoms is still controversial. Some studies reported that mindfulness-based and acceptance meditation interventions were effective for positive symptoms (Chien et al., 2017, 2019; Cramer, Lauche, Haller, Langhorst, & Dobos, 2016; Wang et al., 2016). Our previous study showed that 8 months of MBI could alleviate positive symptoms, such as hallucinations and delusions, in patients with severe schizophrenia (Sheng, Yan, Yang, Yuan, & Cui, 2019). However, in this study, recruited patients with residual negative symptoms had no severe positive symptoms, and more than one-third had no positive symptoms. Therefore, there was little room for positive symptoms to improve. In addition, 6 weeks of MBI may be too short of a duration to impact patients’ remaining delusions or hallucinations.

In this study, we found no significant effect of MBI on depression and anxiety symptoms. However, the significant decrease of the total score of SCL-90 still indicated the initial effect of 6-week MBI on the overall physical and mental symptoms of schizophrenia patients. A systematic review and meta-analysis has shown that MBI can improve the depressive symptoms of patients on the schizophrenia spectrum (Jansen et al., 2020). A possible mechanism for the improvement of emotional state could be related to the increase of mindfulness and self-empathy, as well as the decrease of repetitive negative thinking (rumination and anxiety) (Gu, Strauss, Bond, & Cavanagh, 2015). In this study, the depression and anxiety symptoms of the patients were mild, which may lead to the MBI effect cannot be well presented. In the future, schizophrenia patients with residual depression and/or anxiety symptoms should be recruited to further verify the effects of MBI on these dimensions.

There were several limitations in this study. Firstly, the 6-week follow-up period was too short, and MBI may take longer to play a more significant role, especially for cognitive impairment. In the future, more long-term follow-up studies should be conducted. Secondly, the Five Facet Mindfulness Questionnaire (FFMQ) is a self-assessment scale to measures of individual perceived levels of mindfulness. In this study, we have used FFMQ to evaluate the quality of mindfulness practice, but many chronic patients with schizophrenia have not completed this assessment. Therefore, FFMQ data were not analyzed in this study. Thirdly, the influences of different antipsychotics could not be completely eliminated. Future studies subdividing antipsychotics should be conducted to minimize the interference of different effects of drugs on the results.

## Conclusion

In conclusion, our results show that MBI can help improve clinical symptoms, such as negative symptom, general psychopathology symptom, and cognitive impairment in schizophrenia patients with residual negative symptoms. Our study suggests that MBI can be applied to clinical practice, especially when treating chronic schizophrenia patients who have residual negative symptoms. However, our results need to be further validated by future studies with a larger sample size and long-term intervention.

## Data Availability

The data that support the findings of this study are available from the corresponding author Donghong Cui upon reasonable request.
